# Dual path mechanism of promoting classical furniture and customer responses: From the perspective of empathy

**DOI:** 10.3389/fpsyg.2022.999631

**Published:** 2022-09-27

**Authors:** Jiajun Cai, Lixia Yu

**Affiliations:** ^1^School of Art and Design, Shenzhen University, Shenzhen, China; ^2^School of Economics and Management, Nanchang Institute of Science and Technology, Nanchang, China

**Keywords:** classical furniture, furniture brand Tanjuyuan, practical empathy, cultural empathy, customer response

## Abstract

The correlation between empathy and customer responses may be a key to solve the problem of classical furniture advertising design. To explore the relationship between empathy and consumer purchasing response, this study proposes a model of dual path mechanism of empathy influencing consumer purchase intentions in classical furniture through advertising design related to furniture brand Tanjuyuan. The results not only prove the hypotheses, but also indicate that: (1) cultural empathy and empathy fusion have a more significant impact on consumers’ purchase intention than practical empathy; (2) cultural empathy plays a dominant role in influencing consumers’ purchase intention; (3) empathy fusion is a key mediator between cultural empathy and practical empathy in influencing consumers’ purchase intention. These findings provide issues for subsequent research from various perspectives, such as enhancing the practical perceptions of consumers of classical furniture products, cultural value perceptions, and the interdisciplinary application of empathy.

## Introduction

Empathy may be a key to the design and promotion of classical furniture advertising. Classical furniture advertisement is usually designed to change consumers’ original cognition of classical furniture products, control consumers’ emotions, and further achieve a change in consumer behavioral decisions. Advertising designers should recognize that consumer behaviors are influenced by the design and promotion of their advertisement. With the coming of consumer experience-centered era, factors that influence or determine consumer behavior decisions are also gradually changing (Deng et al., 2022; Li et al., 2022; Wang et al., 2022). When purchasing classical furniture products, consumers shift their attention from product attribute to value attribute, and the increasing consumption awareness makes consumers pay more attention to the emotional experience in shopping process. However, existing advertising promotions still have not realized the change of focus from product to people. Furthermore, the decision-making process of consumer, considered as the black box of consumer psychology, reflects consumers’ mental activity and has a key impact on marketing activities (Shaw and Bagozzi, 2018; Li and Katsumata, 2020; Li et al., 2021a,b). Therefore, the key issue in the design and promotion of classical furniture advertising is how to create content that could stimulate consumers’ purchase intention.

In recent years, some scholars have found that empathy can have a positive impact on consumers’ purchase intention, and then promote their consumptions (Gillani et al., 2021; Jing et al., 2022; Yi et al., 2022). Why does empathy affect consumer behavior? This is because empathy and consumer behavioral decision share the same attributes in the psychological context. Empathy can help create a psychological resonance between consumers, which in turn affects consumers’ perception of classical furniture products and further change their behavioral decision. In addition, the empathy fusion in the design and promotion of classical furniture advertising can better meets the emotional needs of consumers and helps the brand achieve value enhancement, as well as perpetuates the vitality of classical furniture products and enhances the brand awareness. To implement the consumer-centered concept in the design and promotion of classical furniture advertising, designers need to study deeply how to give the product a richer cultural connotation, in order to trigger consumer empathy and stimulate their purchase behavior.

In existing research, scholars have conducted relatively abundant research on empathy theory, consumer behavioral decision, and factors that influence consumer behavioral decision in advertising design. However, they have not explored these issues in a unified framework for discussion. There are several key issues that need to be addressed: First, is there a correlation between consumer behavioral decision and empathy? Second, how does advertisement influence consumers’ behavioral decision? Third, how can empathy be used in the design and promotion of classical furniture advertising to influence consumers’ purchasing decision?

In response to above questions, this study employs literature review, theoretical analysis, questionnaire survey and empirical analysis as research methods. This study selects pictures of products of Tanjuyuan, a representative brand in the classical furniture, as the experimental object, and collects data through questionnaire survey with considering the authenticity, validity, and availability of data sources. From theoretical point of view, this study innovatively proposes the possible correlation between consumer behavioral decision and empathy theory, enriches the connotation of empathy theory, broadens the application of empathy theory, and successfully constructs and verifies the dual path model of practical empathy and cultural empathy into consumer purchase decision in the process of classical furniture advertising design promotion. From the practical point of view, the study tested the validity of the empathy scale and the consumer purchase intention scale, pushing the classical furniture advertising design into a new dimension. The study also validates and analyzes the paths through which empathy affects consumers’ purchase intentions, providing complete theoretical support and reliable data on how to apply empathy in the design and promotion of classical furniture advertisements. Finally, this study opens a new perspective of classical furniture products from consumers, and it provides a basis for subsequent research on the of empathy communication for other products.

Based on the above three key issues, this study first makes the theoretical analysis to sort out the commonality between empathy and consumer behavioral decision and explores the mechanism of empathy influencing the consumers’ purchase intention of classical furniture. Second, this study collects the data of consumers’ empathy and purchase intention related to classical furniture advertising through questionnaire survey. Finally, this study applies exploratory factor analysis and validation factor analysis to verify the reliability of the dual path mechanism of empathy affects consumers’ purchase intention.

## Literature review and hypotheses

### Literature review

#### Empathy and consumer behavioral decisions

There is no universal definition of empathy in academia. Tracing its roots, the concept of empathy emerged from psychology and has been enriched by interdisciplinary research. Throughout the research of scholars, empathy refers to the ability of individuals to perceive or imagine the emotional state of others, understand and experience the emotions of others, then make a certain response (Rivas and Husein, 2022). Instead of being an emotion, empathy is a process of experiencing another person’s emotions, emphasizing the ability to resonate with others and having the following three characteristics. First, empathy is objective and fact-based, which has three stages of emotional contagion, perspective taking and empathic concern (Hogan, 1969; Eklund and Meranius, 2021). Second, empathy is heterogeneous. Depending on the target and the situation, there are also significant differences in the strength of empathy generated (Barker et al., 2022). Third, the evolution of empathy could be both positive and negative. Positive empathy is a process of understanding and indirectly sharing others’ positive emotional states, while the negative empathy is a process of understanding and indirectly sharing others’ negative emotional states. Empathy is a genetically rooted unconscious ability that can be either a cognitive outcome or an emotional state. From the perspective of structure, empathy is multidimensional and generally includes affective empathy and cognitive empathy (Gong et al., 2020; Pedersen, 2021; Weisz et al., 2022). Specifically, affective empathy refers to that individuals can produce alternative responses to others’ emotions. It is a bottom-up emotion, which generally follows the Perception-Action model. Cognitive empathy refers to individuals’ ability to recognize others’ emotions and understand others’ views. It is a top-down emotion that involves self-objectification (Ohba and Deen, 2022).

Consumer behavioral decision refers to the process in which consumers are motivated to purchase based on their own needs, form their own cognition, and generate corresponding emotion by perceiving the received product information, engage in purchasing behavior finally (Leung et al., 2022; Sajja, 2022). The process of consumer behavioral decision consists of three aspects: cognitive analysis of products, emotional inspiration, and purchase behavior (Shen and Wang, 2022), with a causal relationship between the first two and the latter one. Among them, consumption cognition is the subconscious memory of products, changes in consumption structure and belief content, whereas consumption emotion is the aesthetic and hedonic feelings experienced by consumers in the process of purchasing (Du and Lin, 2022; Luqman et al., 2017).

Empathy is consistent with consumer behavioral decisions. Empathy and consumer behavior decision making are highly similar in attributes, both belong to psychology; they are also highly compatible in essence, both are a kind of cognition of individuals; and they are structurally identical, both contain cognitive and emotional dimensions. Therefore, it is of practical significance and theoretical value to explore whether empathy theory affects consumers’ purchase decisions.

#### Empathy in the advertising design and promotion of classical furniture

The consumer behavioral decision in the advertising design and promotion of classical furniture refers to a process of consumers selecting, absorbing and processing the received advertising information to form a unique judgment about of classical products, and then generates the consumption cognition and stimulates consumption emotion of classical furniture products, and finally make the purchase decision (Sun et al., 2022). The role of classical furniture advertising design and promotion is to transform consumers’ perceptions and influence their behavior (Anghel, 2022). In the literature of advertising, the information that affect consumer behavioral decision are diverse, not only including explicit visual information that displays the appearance, quality and function of products, but also implicit conceptual information that contains cultural elements such as values and social consensus (Cai, 2017; Majer et al., 2022; Martinčić et al., 2022). Furthermore, according to the means of consumers receiving advertisements, the sources of information can be divided into direct reception and indirect reception (Miliano et al., 2018). Direct reception refers to advertising that presents products directly to consumers in a visual form, including linguistic features, display methods and techniques, which directly affects consumers’ information processing and leads to consumer emotions about classical furniture, ultimately affects consumers’ purchase decision-making (Farshbaf-Geranmayeh et al., 2018). The direct reception is relatively objective, in which the information for consumer behavioral decision is only based on the practical characteristics of the products and personal subjective emotional perception, and consumers will only empathize with the product itself. The indirect reception refers to consumers’ sharing of information through other people’s re-creation of product advertising content (Lee and Lee, 2016), and forming their own unique opinions through empathizing or value judgment. Therefore, the information source of consumer behavioral decision in the advertising design and promotion is mainly the practicality and cultural value of classical furniture products.

Based on the theory of empathy and consumer behavioral decision, this study believes that empathy in the advertising design and promotion of classical furniture refers to the ability of consumers to resonate with other consumers’ attitudes toward classical furniture products both cognitively and emotionally, and to show a desire to buy and share. Since classical furniture has dual attributes of product and culture, and the information source of consumer behavioral decision also has the distinction of explicit visual information and implicit conceptual information, a distinction should be made when analyzing the empathy generated by consumers. Pragmatic empathy based on classical furniture product attributes and explicit visual information, refers to the resonance ability generated by the practical experience of consumers and other consumers in the appearance, quality and safety of classical furniture products. Cultural empathy based on the cultural attributes of classical furniture products and implicit conceptual information, refers to the resonance ability of consumers to resonate with each other in terms of cultural connotations and historical origins of classical furniture products. It is worth emphasizing that the characteristics of pragmatic empathy and cultural empathy have not changed in the process of advertising design and promotion of classical furniture which still retain objectivity, heterogeneity, and differences of evolution trend.

#### Research gaps

Overall, scholars have conducted relatively detailed research on empathy theory, consumer behavioral decision, and advertisement design and promotion. Studies on empathy have defined the concept, discussed the characteristics, and analyzed the structure. The study of consumer behavioral decision making is more generally based on a psychological perspective. Research on advertising design and promotion has mainly focused on the influence of the context, situation and visual design of the advertisement itself on consumer decision making. However, empathy, advertising design and promotion, and consumer behavioral decision making have not been explored in a unified framework. The following points need to be further explored. Firstly, it is known that consumer behavioral decision-making is highly consistent with empathy in terms of attributes, but the correlation between the two needs a further refinement study. Secondly, there are relatively few studies on consumer behavioral decision in the advertising design and promotion of classical furniture, so detailed research needs to be conducted into this field. Finally, solutions are needed to apply the dual path mechanism of practical empathy and cultural empathy to the design and promotion of classical furniture advertisements, in order to achieve more effective influence on consumers’ purchase decisions.

### Research hypotheses

#### Practical empathy and purchase intention

Based on the product attributes of classical furniture, there is a path for practical empathy to affect consumer behavioral decisions. In the advertising design and promotion of classical furniture, the information that affects consumer behavioral decisions is mainly explicit visual information that demonstrates the appearance, quality and function of products (Anderson and Laverie, 2022; Cai, 2022a). Therefore, the research on how the practical empathy affecting purchase intention mainly focuses on four dimensions of product appearance, product quality, product safety and product trust (Chaerudin and Syafarudin, 2021). The appearance of products is the basic factor that affects consumers’ preferences, product quality is the main factor that affects consumers’ purchase intention, product safety is the potential factor that affects consumers’ purchase intention, and product trust is the core factor that affects consumers’ repeated purchase (Veselovská, 2022; Chen et al., 2022). Therefore, in the process of practical empathy, consumers use their imagination and cognition to select their own unique viewpoints on products. Due to the emotional influence of other people’s attitudes towards product appearance, quality, safety and trust, resulting in positive or negative empathic concerns and finally influencing consumers’ purchase intention (Nguyen and Chao, 2021). Therefore, this study proposes the following hypothesis:

*H1:* Practical empathy has a positive effect on the consumers’ purchase intention of classical furniture products.

#### Cultural empathy and purchase intention

Based on the cultural attributes of classical furniture products, there exists a path for cultural empathy to affect consumer behavioral decisions in the advertising design promotion of classical furniture, the information that affects consumer behavioral decisions comes from implicit conceptual information containing many cultural elements such as classical furniture culture, value and social consensus (Cai and Zhou, 2021; Reyes-Menendez et al., 2022). In the process of empathic communication of classical furniture culture, consumers develop cultural perceptions and emotional states similar to other consumers, through alternative experiences of their cultural emotions of classical furniture (Zhao et al., 2021). Therefore, the research on the path of cultural empathy affecting consumers’ purchase intention mainly focuses on cultural elements such as cultural theme, historical origin, craftsmanship and cultural identity of classical furniture products (Swami et al., 2022). Among them, cultural theme is the outermost factor that attracts consumers’ attention, historical origin is the potential factor of consumers’ understanding, craftsmanship is the key element of consumers’ value perception, and cultural identity is the decisive factor of consumers’ empathy and purchase behavior (Jamal and Sharifuddin, 2015). In the generation process of empathy on classical furniture culture, consumers are influenced by others’ evaluation and experience of multi-dimensional cultural elements of classical furniture products, resulting in cultural emotional contagion, combining with their own imagination and cognition of classical furniture culture to make “perspective-taking,” and finally generate sharing or purchasing behaviors based on the results of empathic concerns (Sharifi-Tehrani et al., 2022; Cai, 2022b). Therefore, this study proposes the following hypotheses:

*H2:* Cultural empathy has a positive effect on the consumers’ purchase intention of classical furniture products.

#### Practical empathy, cultural empathy and purchase intention

Practical empathy is the foundation of cultural empathy, and cultural empathy is the superstructure of practical empathy, and there is both a bottom-up and top-down logical relationship between the above two. Consumers buy classical furniture products to meet their consumption needs, which can be based on a lower level of material need arising from the product itself which is the lowest level of needs or a higher level of the spiritual need arising from the product culture (Palumbo et al., 2021). Only when consumers’ practical material needs are satisfied can they seek to satisfy their cultural spiritual needs (Lienert, 2015). The logical sequence of practical empathy followed by cultural empathy provides theoretical support for the emergence of cultural empathy among consumers (Yi et al., 2022). Cultural empathy, influencing practical empathy from top to bottom, reinforces the effect of fusion empathy communication, thereby increasing the purchase intention of consumers (Hyun Park et al., 2017). Practical empathy modifies consumers’ perceptions of the practicality of classical furniture products and stimulates positive emotions about the products from the surface; cultural empathy modifies consumers’ perceptions of the cultural characteristics of classical furniture products and stimulates positive emotions about the products from the inside, and the two interact to form fusion empathy, which ultimately influences consumers’ purchase decisions (Everson et al., 2015). Therefore, this study proposes the following hypotheses:

*H3:* Practical empathy moderating the effect between cultural empathy and the consumers’ purchase intention of classical furniture products.

*H4:* Cultural empathy mediating the relationship between practical empathy affecting and consumers’ purchase intention on classical furniture products.

**Figure 1:**
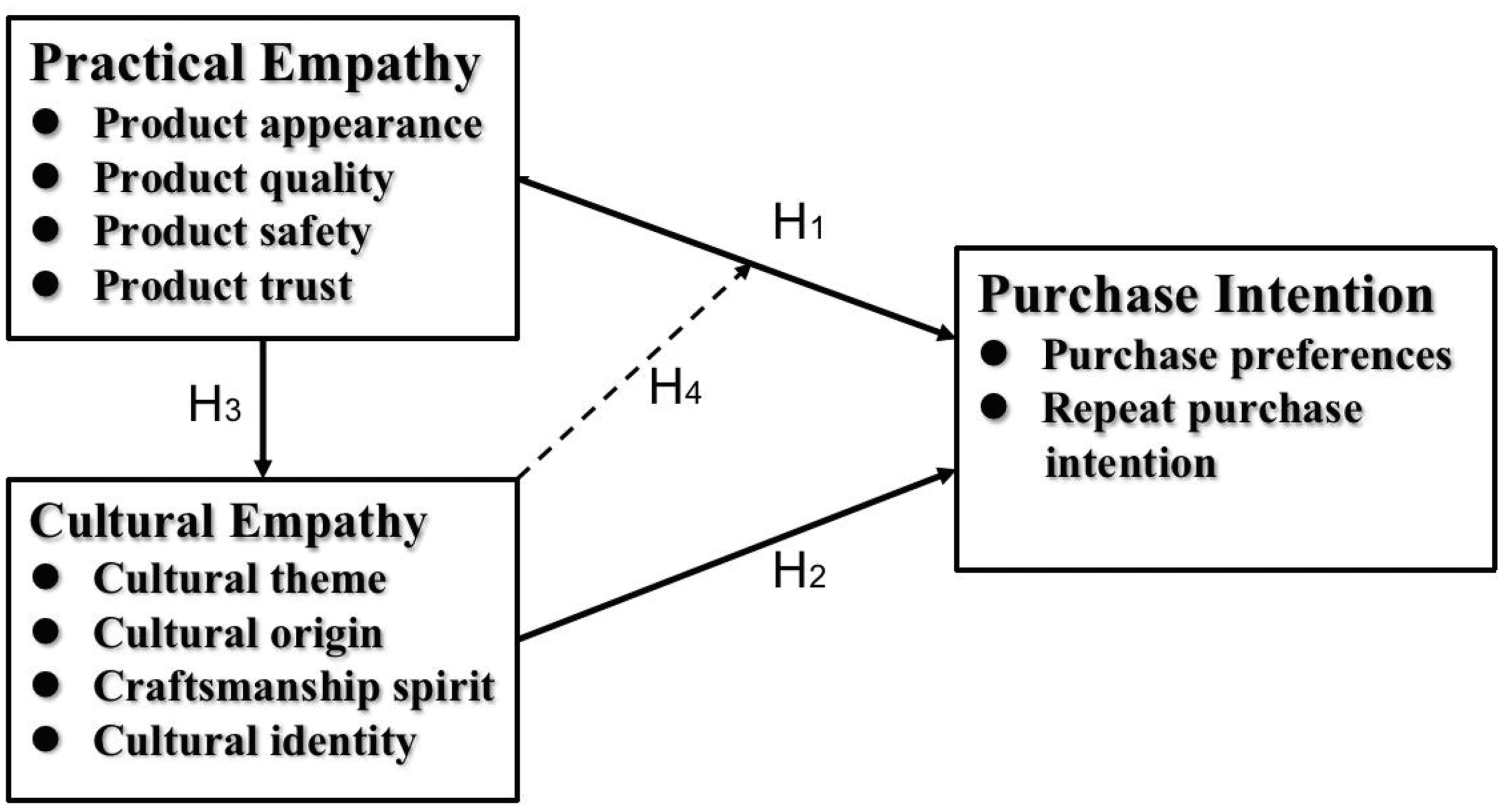
Research framework.

Practical and cultural empathy can independently influence consumers’ purchase intentions, while practical empathy acts on cultural empathy to form a fusion of empathy that will ultimately affect consumers’ consumption behavior decisions as well. The overall paths of empathy affecting consumers are shown in Figure 1.

## Methodology

### Research plan

**Figure 2:**
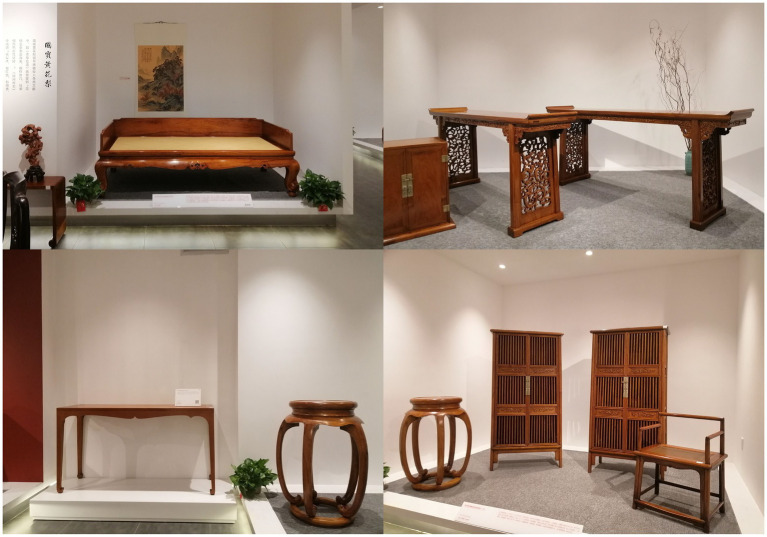
Classical furniture brand of Tanjuyuan. Those photos were provided by Jiajun Cai.

In line with the principles of practical empathy and cultural empathy, this study selected furniture brand Tanjuyuan as the research object and used pictures of its products as the experimental object, as are shown in Figure 2. This study also added to the pictures not only practical information such as product appearance and materials, but also cultural information that can highlight the theme, historical origin, and inherited spirit of furniture products. This study adopted the experimental method of questionnaire survey to verify the hypotheses of empathy paths in the advertising design of classical furniture.

This study constructed an empathy measurement scale for the advertising design promotion of classical furniture, considering the research object, based on the Interpersonal Relationship Indicator Scale (IRI; Ingoglia et al., 2016; Fultz and Bernieri, 2022) and Questionnaire of Cognitive and Affective Empathy (QCAE; Şakir et al., 2021), which are widely used in empathy measurement. To verify the rationality of the empathy scale, the questionnaire data from the pre-study were analyzed for reliability and validity, and the empathy scale was revised and formed for this study. The scale is mainly divided into four parts. The first part is the practical empathy scale, which mainly involves four dimensions of product appearance empathy, product quality empathy, product safety empathy and product trust empathy, with 12 items. The second part is the cultural empathy scale, which mainly includes 12 items of four dimensions related to empathy of cultural theme, cultural origin, craftsmanship and cultural identity. The third part is the integrated empathy scale, which mainly includes three items related to the interaction between practical empathy and cultural empathy. The fourth part is the consumers’ purchase intention scale, including two items of two dimensions including first-time purchase and repeated purchase. Likert 5-point scale was adopted in the questionnaire.

### Data collection

Affected by the epidemic, the questionnaire survey was carried out online, mainly on social platforms such as Wechat, QQ and Weibo from May to June 2022. A total of 360 questionnaires were collected, 348 of them were screened by validity, with an effective rate of 96.7%. Those questionnaires were randomly divided into two parts, 174 copies each, were used for exploratory factor analysis and confirmatory factor analysis, respectively. The basic characteristic variables are as shown in Table 1, which indicate that the proportion of male and female respondents is relatively balanced. Since the audience of classical furniture products is mainly middle-aged and elderly people, the questionnaire was distributed with certain target, leading to the age of the respondents being concentrated in the range above 40 years old. The respondents’ educational background is primarily below college level, and with a monthly income above 6,000 RMB.

**Table 1:** Sample basic characteristic variable statistics.

**Statistics**	**Category**	**Number**	**Proportion**
Gender	Male	164	47.1%
	Female	184	52.9%
Age	Under 18	16	7.5%
	18–29	62	17.8%
	30–39	36	10.3%
	40–49	104	29.9%
	More than 50	120	34.5%
Education Background	Below High School	140	40.2%
	Specialized subject	110	31.6%
	Undergraduate course	68	19.5%
	Above Postgraduate	30	8.7%
Income (RMB)	Under 3,000	88	25.3%
	3,000–6,000	54	15.5%
	6,000–9,000	120	34.5%
	More than 9,000	86	24.7%

### Validity of measurements and model fit testing

This study adopted SPSS24.0 and AMOS24.0 software to analyze the data for the explore and validate the empathy paths in the advertising design and promotion of classical furniture. SPSS24.0 software was used to conduct exploratory factor analysis to verify the reliability of the empathy scale and modify the empathy model. AMOS24.0 was used to conduct goodness-of-fit tests and structural equation analysis of the modified empathy model (e.g., Luqman and Zhang, 2022).

In this study, KMO test value and Bartlett spherical test were performed on the 174 valid samples. KMO value is 0.957, Bartlett spherical test value is 2,574.054, and the significance is <0.001; The reliability analysis is also made, and Cronbach’s *α* value is 0.963. Overall, the reliability and validity of the scale are excellent, which meets the standard of exploratory factor analysis (Luqman et al., 2021; Shaikh et al., 2022; Zhang et al., 2022).

In this study, the covariance matrix is constructed, the factors are extracted by principal component analysis, and rotated by the maximum variance method. After six rotations, the convergence is achieved. The indicators demonstrate a clear four-factor structure, and the total variance interpretation rate is 80.2%. The items were screened according to the principle of item analysis, the final component matrix is as shown in Table 2: factor 1 is practical empathy, which mainly includes seven indicators from a1 to a7; factor 2 is cultural empathy, which mainly includes six indicators from b1 to b6; factor 3 is blend empathy, which mainly includes one indicator of f2; factor 4 is purchase intention, including two indicators of f1 and f3.

**Table 2:** The rotated component matrix

**Outcome measurement**	**Practical empathy**	**Cultural empathy**	**Empathy fusion**	**Purchase intention**
a1 Emotional contagion of product appearance	0.666			
a2 Product quality point of empathic concern	0.524			
a3 Product quality point of view selection	0.785			
a4 Selection of product appearance	0.605			
a5 Emotional contagion of product safety	0.506			
a6 Product safety perspective selection	0.532			
a7 Empathic concerns about product trust	0.591			
b1 Selection of views on cultural themes		0.721		
b2 Cultural themes empathic concerns		0.562		
b3 Cultural origin perspective selection		0.699		
b4 Cultural origin empathic concerns		0.673		
b5 Craftsmanship empathy		0.670		
b6 Empathic concerns about cultural identity		0.592		
f1 Purchase preference				0.585
f2 Empathic interaction			0.716	
f3 Repeated purchase intention				0.629

### Mediation model fit testing

Based on exploratory factor analysis, AMOS24.0 was used to conduct confirmatory factor analysis (CFA) on the remaining 174 valid questionnaires, and the mediation effect of the model was verified by Bootstrap test. The overall fitting results of the model are as shown in Table 3: CMIN/DF is 1.132 (<2), which reveals a good test value; indicators such as GFI, AGFI, CFI, RFI, IFI, NFI and NNFI are all >0.9, suggesting excellent goodness-of-fit; ECVI value is small, RMR is 0.027 (close to 0), which can be seen all indicators show excellent goodness-of-fit. Therefore, the CFA suggests that the empathy path model and factors have excellent goodness-of-fit, and all parameters are valid (Hox and Bechger, 1998).

**Table 3:** Mediation model fit.

**Statistical detection quantity**	**Parameter values**
CMIN/DF	1.132
GFI	0.929
AGFI	0.902
CFI	0.990
RFI	0.907
IFI	0.990
NFI	0.924
NNFI(TLI)	0.988
ECVI	1.122
RMR	0.027

## Results

### Hypothesis test results

Verifying the above hypotheses, it can be obtained that the coefficient of H1 is 0.23, the coefficient of H2 is 0.10, the coefficient of H3 is 0.90, and the coefficient of H4 is 0.63. Therefore, the four hypotheses in this study are supported, and the effect is positive. The detailed information was shown in Table 4.

**Table 4:** Model hypothesis results.

**Research hypothesis**	**SEM**	**Path coefficient**	**Test results**
H1: PE has an effect on PI	PE → IE → PI	0.23	Supported
H2: CE has an effect on PI	CE → PI	0.10	Supported
H3: PE has an effect on CE	PE → CE	0.90	Supported
H4: CE has an effect on PE to PI	CE → IE → PI	0.63	Supported

PE, Practical Empathy; CE, Cultural Empathy; IE, Integrated Empathy; PI, Purchase Intention.

**Figure 3:**
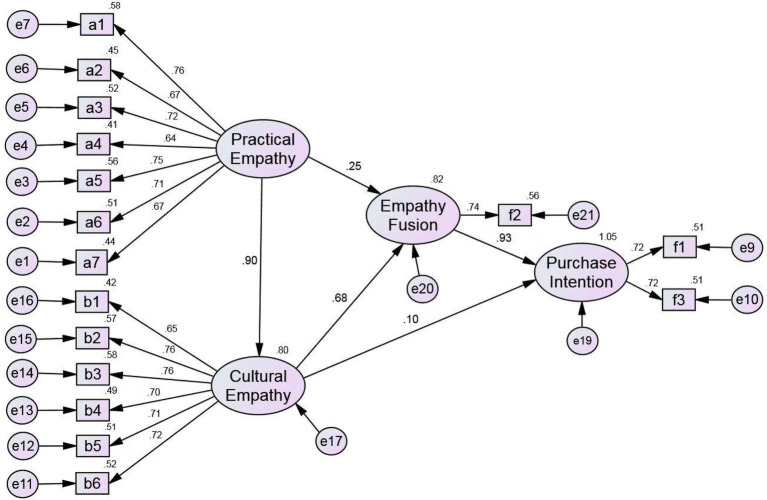
Structure model.

From Table 4, it can be concluded that the coefficients of emotional contagion of product appearance (a1), product quality point of view selection (a3), emotional contagion of product safety (a5), and product safety perspective selection (a6) range from 0.7 to 0.8; product quality point of empathic concern (a2), selection of product appearance (a4), and empathic concern about product trust (a7) range from 0.6 to 0.7, showing a high significance; In the items of cultural empathy and practical empathy, cultural theme empathic concerns (b2), cultural origin perspective selection (b3), cultural origin empathic concern (b4), craftsmanship empathic concern (b5) and empathic concern about cultural identity (b6) are >0.7, except the coefficients of selection of views on cultural themes (b1) which is between 0.6 and 0.7, showing a high significance; In terms of empathy fusion, the empathic interaction (f2) coefficient is 0.74, which is highly significant; in terms of purchase intention, the coefficient of purchase preference (f1) and repeated purchase intention (f3) are both 0.72, with a higher significant level. Overall, each factor loading shows a valid level, which can effectively reflect the correlation between practical empathy, cultural empathy, empathy fusion and purchase intention. These factors constitute a practical-cultural dual empathy model as shown in Figure 3.

### Research conclusions

According to the above research, the factors affecting consumers’ purchase intention mainly include practical empathy, cultural empathy, and empathy fusion. Among them, cultural empathy plays a leading role in affecting the consumers’ purchase intention. Besides, empathy fusion is the pivotal mediator for cultural empathy to realize its role in the effect of practical empathy on consumers’ purchase intention. Compared with the direct effect of practical empathy, the indirect influence of cultural empathy and empathy fusion on consumers’ purchase intention is more significant. Therefore, in the design and promotion of classical furniture advertising, the paths through which practical and cultural empathy affect consumers’ purchase intentions are divided into four stages: first, cultural empathy directly affects consumers’ purchase intentions; second, cultural empathy indirectly affects consumers’ purchase intentions by influencing empathy fusion; third, practical empathy affects consumers’ purchase intentions by influencing empathy fusion; fourth, practical empathy affects consumers’ purchase intentions by influencing cultural empathy and then empathy fusion.

## Discussions

### Theoretical implications

First, this study verifies that consumer behavioral decision can effectively interconnect with empathy theory. Factors such as empathy, advertising design and promotion, and consumer behavioral decision have been explored within a unified framework, and a dual path model of practical and cultural empathy influencing consumer decision in the process of classical furniture advertising design and promotion is constructed. Furthermore, based on the theory of consumer behavioral decision, this study introduces the explicit visual elements related to the four dimensions such as product appearance, product quality, product safety and product trust in the advertising design of classical furniture into the practical empathy scale. Finally, factor analysis verifies the connotation and the rationality of the practical empathy scale.

Second, this study enriches the connotation of empathy theory and broadens its application in the advertising design and promotion of classical furniture. Based on the dual attributes of product and culture of classical furniture, this study redefined the theory of empathy from the perspective of both practical empathy and cultural empathy. Moreover, practical empathy and cultural empathy are introduced into the process of advertising design promotion of classical furniture, which enriches the research of contributing factors of consumer behavioral decision on classical furniture products.

Third, this study constructs and verifies a dual-path model for how to utilize practical empathy and cultural empathy to affect consumers’ purchase decision in advertising design and promotion of classical furniture. In the process of influencing consumer behavioral decision, practical empathy and cultural empathy enhance consumers’ purchase intention of classical furniture products. In addition, empathic fusion plays a pivotal mediating role in the process of practical empathy affecting consumers’ purchase intention, which is the core of positively influencing the purchase intention of classical furniture consumers.

### Practical implications

First, it is necessary to improve the quality of classical furniture products and optimize advertising design and promotion to enhance consumers’ practical perceptions. According to the results of structural equation model analysis, it is clear that practical empathy is the starting point in all four paths of consumer purchase intention, which shows that practical empathy is the basis for the influence of empathy theory to influence consumer behavior decisions. In fact, the effect of practical empathy on consumers’ purchase intention is not significant, but indirectly affects consumers’ behavioral decisions through cultural empathy and empathy fusion. Therefore, the quality of classical furniture products, as the most basic element, should play its role well and update classical furniture products and advertising design according to consumers’ needs and aesthetics in a timely manner, so as to provide consumers with a good consumption experience. Additionally, due to the obvious product attribute and characteristics of classical furniture, the advertising design and promotion should also focus on product appearance, quality and safety. These aspects provide guarantees for consumers’ practical perception and improve their purchase intention.

Second, it is necessary to enrich the cultural connotation of classical furniture to ensure consumers’ good cultural value perception. According to the results of structural equation model analysis, cultural empathy not only directly affects consumers’ purchase intention, but also indirectly affects consumers’ purchase intention through the role of empathy fusion. The effect of cultural empathy is related to the effect of cultural cognition and emotional cognition of classical furniture consumers, so classical furniture manufacturers and advertising producers, as the creators of classical furniture product culture, should take more efforts to improve the quality of product cultural content. The focus of cultural themes and the depth of product origin in advertising design of classical furniture are the basic cultural content requirements to enhance the consumers’ purchase intention. Besides, the craftsmanship contained in the production technology is utilized to achieve the consumers’ recognition of the cultural value of classical furniture products and realize consumers’ deep and immersive cultural empathy. The consumers’ cultural consumption needs should be well satisfied by ensuring their perception of the cultural value of classical furniture and creating a brand of classical furniture with cultural connotation.

Third, it is necessary to build the interconnection between consumers’ practical and cultural empathy to enhance consumers’ purchase intention. According to the results of structural equation model analysis, empathy fusion is one of the important factors that affect consumers’ purchase intention. In the process of consumption, consumers not only meet their needs of products, but also meet their pursuit of cultural spirit and emotional experience. Therefore, in the advertising design of classical furniture products, we should actively integrate practical empathy into cultural empathy to strengthen the interaction of consumers’ cognition and emotional experience of classical furniture products and culture to further enhance consumers’ purchase intention.

## Conclusion

This study innovatively adopts the dual perspectives of practicality and culture to construct the mechanism of empathy effect on consumers’ purchase intention in the design and promotion of classical furniture advertisements. In the meanwhile, it verifies the reliability through an empirical analysis of the structural equation model. It also reveals that there are four main paths for practical empathy and cultural empathy to affect consumers’ purchase intention, among which the empathy fusion formed by the interaction of practical empathy and cultural empathy is the pivot in the mechanism. However, this study also has some limitations, which need to be further improved in the follow-up studies. First, the questionnaires were only distributed to targeted customers of classical furniture products within a specific age range, there may be some risks in the representativeness of the data. Second, factors that affect consumers’ purchase decision are relatively diverse. This study explored only those from the perspective of consumers’ psychology, so the conclusions may not be comprehensive enough. Third, the channels and methods of advertising design and promotion of classical furniture products may also affect consumers’ purchase intention, which can be explored further in subsequent research.

## Data availability statement

The raw data supporting the conclusions of this article will be made available by the authors, without undue reservation.

## Author contributions

JC contributed to the design of the framework, the collection of primary materials, the framework development, and the revision of the total work. LY contributed to the empirical work, the analysis of the results, and the writing of the first draft. Both authors contributed to the article and approved the submitted version.

## Funding

This study is supported by Fund Project of the Humanities and Social Sciences Research Program of the Ministry of Education (no. 19YJC760002); Shenzhen Scientific-research Start-up Fund for High-Level Talent (no. 827/000572); Shenzhen Educational Science Planning Project (no. ybfz18037).

## Conflict of interest

The authors declare that the research was conducted in the absence of any commercial or financial relationships that could be construed as a potential conflict of interest.

## Publisher’s note

All claims expressed in this article are solely those of the authors and do not necessarily represent those of their affiliated organizations, or those of the publisher, the editors and the reviewers. Any product that may be evaluated in this article, or claim that may be made by its manufacturer, is not guaranteed or endorsed by the publisher.

## References

[ref1] Anderson K. C. Laverie D. A. (2022). In the consumers’ eye: a mixed-method approach to understanding how VR-content influences unbranded product quality perceptions. J. Retail. Consum. Serv. 67:102977. doi: 10.1016/j.jretconser.2022.102977

[ref2] Anghel M. A. (2022). Modeling consumer behavior–psychological paradigm. J. Educ. Soc. Multiculturalism 3, 203–210. doi: 10.2478/jesm-2022-0013

[ref3] Barker M. E. Crowfoot G. King J. (2022). Empathy development and volunteering for undergraduate healthcare students: a scoping review. Nurse Educ. Today 116:105441. doi: 10.1016/j.nedt.2022.105441 35751985

[ref5] Cai J. J. (2017). The necessity of rediscovering aesthetics and design theories in confucianism. Art Educ. 308, 120–125.

[ref6] Cai J. J. (2022a). Study on the functional adaptability of Ming-style furniture and the “body & function” theory in its design. Furniture Interior Des. 29, 48–53. doi: 10.16771/j.cn43-1247/ts.2022.01.010

[ref7] Cai J. J. (2022b). Discussion on the social adaptability of Ming-style furniture and the theory of “heaven & earth/yin & yang” in its modeling design. Furniture Interior Des. 29, 68–74. doi: 10.16771/j.cn43-1247/ts.2022.06.011

[ref8] Cai J. J. Zhou Y. L. (2021). Expounding on the cultural adaptability of Ming style furniture by paying attention to the difference between Ming and Qing dynasty. Art Des. 2, 98–104. doi: 10.16824/j.cnki.issn10082832.2021.11.025

[ref300] Chaerudin S. M. Syafarudin A. (2021). The effect of product quality, service quality, price on product purchasing decisions on consumer satisfaction. Ilomata Int. J. Tax Accounting 2, 61–70.

[ref9] Chen H. Chen H. Tian X. (2022). The impact of social shopping feature richness on buying intention: a product perspective. Internet Res. 32, 1378–1400. doi: 10.1108/INTR-05-2021-0313

[ref11] Chui H. Li X. Luk S. (2022). Therapist emotion and emotional change with clients: effects on perceived empathy and session quality. Psychotherapy 35771517. doi: 10.1037/pst0000442 35771517

[ref12] Deng J. Liu J. Yang T. Duan C. (2022). Behavioural and economic impacts of end-user computing satisfaction: innovative work behaviour and job performance of employees. Comput. Hum. Behav. 136:107367. doi: 10.1016/j.chb.2022.107367

[ref13] Du G. Lin Y. (2022). Brand connection and entry in the shopping mall ecological chain: evidence from consumer behavior big data analysis based on two-sided markets. J. Clean. Prod. 364:132663. doi: 10.1016/j.jclepro.2022.132663

[ref14] Eklund J. H. Meranius M. S. (2021). Toward a consensus on the nature of empathy: a review of reviews. Patient Educ. Couns. 104, 300–307. doi: 10.1016/j.pec.2020.08.022, PMID: 32888755

[ref15] Everson N. Levett-Jones T. Lapkin S. Pitt V. Van der Riet P. Rossiter R. (2015). Measuring the impact of a 3D simulation experience on nursing students’ cultural empathy using a modified version of the Kiersma-Chen empathy scale. J. Clin. Nurs. 24, 2849–2858. doi: 10.1111/jocn.12893, PMID: 26178187

[ref16] Farshbaf-Geranmayeh A. Rabbani M. Taleizadeh A. A. (2018). Channel coordination with cooperative advertising considering effect of advertising on willingness to pay. J. Optim. Theory Appl. 176, 509–525. doi: 10.1007/s10957-018-1217-5

[ref17] Fultz A. A. Bernieri F. J. (2022). Observer descriptions of the empathic person: a look at the Davis IRI and Hogan empathy scales. J. Soc. Psychol. 162, 26–40. doi: 10.1080/00224545.2021.1985416, PMID: 34850660

[ref18] Gillani A. Kutaula S. Leonidou L. C. Christodoulides P. (2021). The impact of proximity on consumer fair trade engagement and purchasing behavior: the moderating role of empathic concern and hypocrisy. J. Bus. Ethics 169, 557–577. doi: 10.1007/s10551-019-04278-6

[ref19] Gong M. Xu M. Luqman A. Yu L. Masood A. (2020). Understanding the role of individual differences in mobile SNS addiction. Kybernetes 49, 3069–3097. doi: 10.1108/K-05-2019-0367

[ref20] Hogan R. (1969). Development of an empathy scale. J. Consult. Clin. Psychol. 33, 307–316. doi: 10.1037/h0027580 4389335

[ref21] Hox J. J. Bechger T. M. (1998). An introduction to structural equation modeling. Family Sci. Rev. 11, 354–373.

[ref22] Hyun Park S. Seon Shin W. Hyun Park Y. Lee Y. (2017). Building a new culture for quality management in the era of the fourth industrial revolution. Total Qual. Manag. Bus. Excell. 28, 934–945. doi: 10.1080/14783363.2017.1310703

[ref23] Ingoglia S. Lo Coco A. Albiero P. (2016). Development of a brief form of the interpersonal reactivity index (B–IRI). J. Pers. Assess. 98, 461–471. doi: 10.1080/00223891.2016.1149858, PMID: 27050826

[ref24] Jamal A. Sharifuddin J. (2015). Perceived value and perceived usefulness of halal labeling: the role of religion and culture. J. Bus. Res. 68, 933–941. doi: 10.1016/j.jbusres.2014.09.020

[ref25] Jing K. Qi M. Mei Y. Chen L. (2022). The impact of empathy with nature on green purchase behavior: an ERP study. Neurosci. Lett. 784:136745. doi: 10.1016/j.neulet.2022.136745, PMID: 35718238

[ref27] Lee J. Lee Y. (2016). The impact of emotional appeals in fair trade apparel advertisements-the interaction effect of advertising channel and the mediation effect of PCE. J. Korean Soc. Costume 66, 49–65. doi: 10.7233/jksc.2016.66.5.049

[ref28] Leung W. K. Chang M. K. Cheung M. L. Shi S. (2022). Understanding consumers’ post-consumption behaviors in C2C social commerce: the role of functional and relational customer orientation. Internet Res. 32, 1131–1167. doi: 10.1108/INTR-11-2020-0664

[ref29] Li X. Katsumata S. (2020). The impact of multidimensional country distances on consumption of specialty products: a case study of inbound tourists to Japan. J. Vacat. Mark. 26, 18–32. doi: 10.1177/1356766719842280

[ref30] Li X. Ouyang Z. Zhang Q. Shang W. L. Huang L. Wu Y. (2022). Evaluating food supply chain emissions from Japanese household consumption. Appl. Energy 306:118080. doi: 10.1016/j.apenergy.2021.118080

[ref31] Li X. Wirawan D. Li T. Yuan J. (2021b). Behavioral changes of multichannel customers: their persistence and influencing factors. J. Retail. Consum. Serv. 58:102335. doi: 10.1016/j.jretconser.2020.102335

[ref32] Li X. Wirawan D. Ye Q. Peng L. Zhou J. (2021a). How does shopping duration evolve and influence buying behavior? The role of marketing and shopping environment. J. Retail. Consum. Serv. 62:102607. doi: 10.1016/j.jretconser.2021.102607

[ref33] Lienert A. (2015). Change of culture or culture of change? Introducing a path-agency-culture (PAC) framework to servitization research. Procedia CIRP 30, 353–358. doi: 10.1016/j.procir.2015.02.094

[ref34] Luqman A. Cao X. Ali A. Masood A. Yu L. (2017). Empirical investigation of Facebook discontinues usage intentions based on SOR paradigm. Comput. Hum. Behav. 70, 544–555. doi: 10.1016/j.chb.2017.01.020

[ref35] Luqman A. Talwar S. Masood A. Dhir A. (2021). Does enterprise social media use promote employee creativity and well-being? J. Bus. Res. 131, 40–54. doi: 10.1016/j.jbusres.2021.03.051

[ref36] Luqman A. Zhang Q. (2022). Explore the mechanism for seafarers to reconnect with work after post-pandemic psychological distress (PAPIST19): the moderating role of health-supporting climate. Ocean Coastal Manage. 223:106153. doi: 10.1016/j.ocecoaman.2022.106153 PMC9464103 36119850

[ref37] Majer J. M. Henscher H. A. Reuber P. Fischer-Kreer D. Fischer D. (2022). The effects of visual sustainability labels on consumer perception and behavior: a systematic review of the empirical literature. Sustainable Prod. Consumption 33, 1–14. doi: 10.1016/j.spc.2022.06.012

[ref38] Martinčić M. Vuković D. Hunjet A. (2022). Consumer responses to selected activities: price increases, lack of product information and numerical way of expressing product prices. J. Risk Financ. Manage. 15:255. doi: 10.3390/jrfm15060255

[ref39] Miliano C. Margiani G. Fattore L. De Luca M. A. (2018). Sales and advertising channels of new psychoactive substances (NPS): internet, social networks, and smartphone apps. Brain Sci. 8:123. doi: 10.3390/brainsci8070123, PMID: 29966280 PMC6071095

[ref40] Nguyen X. Chao C. C. (2021). Revenge consumption, product quality, and welfare. Int. Rev. Econ. Finance 76, 495–501. doi: 10.1016/j.iref.2021.05.007

[ref41] Ohba A. Deen K. (2022). Acquisition of empathy in child Japanese. Lang. Acquis. 29, 260–295. doi: 10.1080/10489223.2021.2017439

[ref42] Palumbo M. D. Imperio M. Tucci V. Cefola M. Pace B. Santamaria P. (2021). Sensor-based irrigation reduces water consumption without compromising yield and postharvest quality of soilless green bean. Agronomy 11:2485. doi: 10.3390/agronomy11122485

[ref500] Pedersen C. L. (2021). Empathy-based marketing. Psychol Market. 38, 470–480.

[ref43] Reyes-Menendez A. Palos-Sanchez P. Saura J. R. Santos C. R. (2022). Revisiting the impact of perceived social value on consumer behavior toward luxury brands. Eur. Manag. J. 40, 224–233. doi: 10.1016/j.emj.2021.06.006

[ref44] Rivas D. F. Husein S. (2022). Empathy, persuasiveness and knowledge promote innovative engineering and entrepreneurial skills. Educ. Chem. Eng. 40, 45–55. doi: 10.1016/j.ece.2022.05.002

[ref45] Sajja P. S. (2022). Hybrid genetic fuzzy system for modeling consumer behavior. Int. J. Bus. Intell. Res. 13, 1–15. doi: 10.4018/IJBIR.301231

[ref46] Şakir G. I. C. A. Büyükavşar A. Iyisoy M. S. Güleç H. (2021). Psychometric properties of questionnaire of cognitive and affective empathy (QCAE): reliability and factor analysis study in Turkish sample. Arch. Neuropsychiatry 58:228. doi: 10.29399/npa.27248 PMC8419731 34526847

[ref47] Shaikh A. A. Lakshmi K. S. Tongkachok K. Alanya-Beltran J. Ramirez-Asis E. Perez-Falcon J. (2022). Empirical analysis in analysing the major factors of machine learning in enhancing the e-business through structural equation modelling (SEM) approach. Int. J. Syst. Assur. Eng. Manage. 13, 681–689. doi: 10.1007/s13198-021-01590-1

[ref48] Sharifi-Tehrani M. Seyfi S. Zaman M. (2022). At the intersection of tourism social entrepreneurship and empathy: development and validation of an empathy scale. J. Bus. Res. 141, 433–447. doi: 10.1016/j.jbusres.2021.11.041

[ref49] Shaw S. D. Bagozzi R. P. (2018). The neuropsychology of consumer behavior and marketing. Consum. Psychol. Rev. 1, 22–40. doi: 10.1002/arcp.1006

[ref50] Shen M. Wang J. (2022). The impact of pro-environmental awareness components on green consumption behavior: the moderation effect of consumer perceived cost, policy incentives, and face culture. Front. Psychol. 13:580823. doi: 10.3389/fpsyg.2022.580823 35795420 PMC9252608

[ref51] Sun W. Yuan M. Zhang Z. (2022). Promoting consumer adoption of electric vehicles from a standard-information-behavior perspective. Information 13:291. doi: 10.3390/info13060291

[ref52] Swami N. S. Mahal B. A. Dee E. C. (2022). Looking beyond the western lens: how culture and identity influence perceptions of empathy in patient-clinician relationships. Cancer 128, 1545–1546. doi: 10.1002/cncr.34075, PMID: 34962644

[ref53] Veselovská L. (2022). Dual quality of products in Europe: a serious problem or a marketing opportunity? Total Qual. Manag. Bus. Excell. 33, 1146–1163. doi: 10.1080/14783363.2021.1940929

[ref54] Wang Y. Liu Y. Huang L. Zhang Q. Gao W. Sun Q. (2022). Decomposition the driving force of regional electricity consumption in Japan from 2001 to 2015. Appl. Energy 308:118365. doi: 10.1016/j.apenergy.2021.118365

[ref55] Weisz E. Chen P. Ong D. C. Carlson R. W. Clark M. D. Zaki J. (2022). A brief intervention to motivate empathy among middle school students. J. Exp. Psychol. 35737526. doi: 10.1037/xge0001249 35737526

[ref56] Yi K. Li Y. Chen J. Yu M. Li X. (2022). Appeal of word of mouth: influences of public opinions and sentiment on ports in corporate choice of import and export trade in the post-COVID-19 era. Ocean Coastal Manage. 225:106239. doi: 10.1016/j.ocecoaman.2022.106239 PMC9700815 36467315

[ref57] Zhang Q. Gao B. Luqman A. (2022). Linking green supply chain management practices with competitiveness during Covid 19: the role of big data analytics. Technol. Soc. 70:102021. doi: 10.1016/j.techsoc.2022.102021 36090699 PMC9439874

[ref58] Zhao Q. Neumann D. L. Yan C. Djekic S. Shum D. H. (2021). Culture, sex, and group-bias in trait and state empathy. Front. Psychol. 12:561930. doi: 10.3389/fpsyg.2021.561930, PMID: 33995162 PMC8113867

